# The Impact of Long-Term Online Learning on Internet Addiction Symptoms among Depressed Secondary School Students: Insights from a Cross-Panel Network Analysis

**DOI:** 10.3390/bs13070520

**Published:** 2023-06-21

**Authors:** Yanqiang Tao, Qihui Tang, Xinyuan Zou, Shujian Wang, Zijuan Ma, Xiangping Liu, Liang Zhang

**Affiliations:** 1 Faculty of Psychology, Beijing Normal University, Beijing 100875, China; 2 Beijing Key Laboratory of Applied Experimental Psychology, National Demonstration Center for Experimental Psychology Education, Beijing 100875, China; 3 School of Psychology, South Normal University, Guangzhou 510631, China; 4 College Students’ Mental Health Education Center, Northeast Agricultural University, Harbin 150030, China

**Keywords:** online learning, internet addiction, depression, secondary school students, network analysis

## Abstract

Background: The COVID-19 pandemic and the shift to online learning have increased the risk of Internet addiction (IA) among adolescents, especially those who are depressed. This study aims to identify the core symptoms of IA among depressed adolescents using a cross-lagged panel network framework, offering a fresh perspective on understanding the interconnectedness of IA symptoms. Methods: Participants completed the Internet addiction test and the Patient Health Questionnaire-9. A total of 2415 students were initially included, and after matching, only 342 students (a cutoff score of 8) were retained for the final data analysis. A cross-lagged panel network analysis was conducted to examine the autoregressive and cross-lagged trajectories of IA symptoms over time. Results: The incidence rate of depression rose remarkably from 14.16% (N = 342) to 17.64% (N = 426) after the four-month online learning. The symptom of “Anticipation” exhibited the highest out-expected influence within the IA network, followed by “Stay online longer” and “Job performance or productivity suffer”. Regarding the symptom network of depression, “Job performance or productivity suffer” had the highest in-expected influence, followed by “Life boring and empty”, “Snap or act annoyed if bothered”, “Check email/SNS before doing things”, and “School grades suffer”. No significant differences were found in global network strength and network structure between waves 1 and 2. Conclusion: These findings prove the negative effects of online learning on secondary students’ mental health and have important implications for developing more effective interventions and policies to mitigate IA levels among depressed adolescents undergoing online learning.

## 1. Introduction

Internet addiction (IA) is defined as a psychological dependence on the Internet, regardless of the type of activity once logged on [1]. Specifically, it refers to the inability to control one’s Internet usage, leading to negative influences in various areas of life, such as psychological, social, academic, and work-related difficulties [2]. The widespread use of the Internet is generally seen as a positive development due to its ability to provide remote access to vast amounts of information and facilitate connections with others. However, it is important to note that excessive Internet usage, especially spending unreasonable amounts of time spent on social networking [3], video games [4], or other non-learning activities, can contribute to the development of IA [5]. This is a matter of great concern, particularly among adolescents [6]. During puberty, adolescents undergo rapid development in cognitive ability, emotion regulation ability, and self-perception [7], rendering them particularly vulnerable to IA [8]. Notably, IA can bring much impairment to adolescents’ development, including damaging adolescents’ life quality [9], increasing interpersonal conflicts [10], and leading to poorer academic achievements [11].

A meta-analysis has demonstrated a continual increase in the prevalence rate of IA in the new generations [12], with the trend exacerbating during the pandemic [13]. Notably, the prevalence rate of IA among Chinese adolescents reaches 13.4% [14]. Additionally, another study investigating adolescents’ internet use behavior reveals that only half of the adolescents maintained proper Internet use during the pandemic, and 26.64% were addicted during this period, while 5.28% developed IA [15]. The COVID-19 pandemic necessitated quarantine policies to curb the rapid spread of the virus, leading to a significant shift to online courses for a substantial number of adolescents. The reduced parental supervision in this context allowed most adolescents to access the Internet freely, increasing their risk of experiencing IA. Consequently, adolescents face an elevated susceptibility to IA due to the circumstances imposed by the pandemic.

Previous studies have established the association between IA and several mental health problems among adolescents, such as sleep disturbance [16], social anxiety [17], and substance abuse [18]. Notably, depression is consistently reported as the most prevalent mental health problem associated with IA [6,19,20] and a significant factor contributing to suicidal ideation [21]. A recent study has clarified the bidirectional relationship between depression and IA among Chinese adolescents, suggesting that the tendency towards depression may predict the occurrence of IA, while IA also largely predicts the onset of depression [22]. However, the mechanisms underlying the relationship between depression and IA still require further exploration.

According to cognitive emotion regulation theory [23], individuals with depression may utilize the Internet to regulate their emotions. They may attempt to modify or regulate their emotional state through online activities and content to alleviate depressive symptoms [24]. Moreover, research findings have also indicated that external-dysfunctional, internal-dysfunctional, and internal-functional emotion regulation significantly predicted IA [25]. Additionally, the social cognitive theory posits that an individual’s thoughts, beliefs, and interpretations can influence their emotions and behaviors [26]. Individuals experiencing depression may hold negative self-perceptions and view social interactions unfavorably. Consequently, they may be more inclined to seek social connections and a sense of belonging online, perceiving such interactions as easier, less critical, and generating fewer negative emotions [27]. This aligns with the theory of compensatory Internet use [28], which suggests that individuals may employ the Internet as a maladaptive coping mechanism for real-world challenges. A cross-sectional study revealed that two-thirds of students reported symptoms of anxiety and depression while engaging in online learning [29].

One undeniable fact is that numerous studies have attempted to uncover how depressed youth may utilize the Internet to fulfill unmet psychological needs in their offline lives [30,31,32], thereby implying the potential for this coping mechanism to foster long-term dependence on the Internet and perpetuate an inescapable vicious cycle. However, despite the Internet becoming a source of comfort and fulfillment for individuals with depression, there is a scarcity of research specifically addressing the disentanglement of Internet addiction symptoms among depressed youth. This hinders our understanding of the intricate relationship between depression and Internet addiction. Former studies have revealed that a loss of interest in communicating with real people is closely related to depression symptoms [33], and adolescents with IA are at a higher risk of suffering from depression [34]. Therefore, interventions targeting Internet addiction may effectively prevent the new incidence of depression or alleviate the symptoms of depression.

From the traditional perspective, mental disorders have been conceptualized as discrete entities [35]. Negative life events can act as triggers for these disorders, and in turn, the disorders can manifest through various symptoms. For example, the loss of a loved one may lead to depression, accompanied by symptoms such as fatigue, anhedonia, guilt, and even suicidal ideation. While the traditional perspective allows for efficient diagnosis of different psychological disorders, it fails to explain the exacerbation of symptoms by other symptoms. To better understand the interplay between symptoms, a new approach called network analysis has emerged. In this approach, mental disorders are viewed as clusters of interconnected symptoms, where one symptom may trigger or influence other symptoms [36]. For example, feeling bored and empty without the Internet may lead to anticipation of future online activities [37]. The network’s nodes refer to diverse symptoms, and the edges refer to the connections between two symptoms [38]. Nodes with high centrality indicate that these symptoms can greatly influence other symptoms, and the strongest edges may help reveal the underlying mechanism of mental illness [36]. Therefore, network analysis allows researchers to identify the most influential symptoms and provide more accurate targets for designing effective interventions.

A study classified participants into the IA group and the non-IA (NIA) group and found that the symptom network of the IA group is significantly different from that of the NIA group [39]. This study has clarified that “fear about boredom if online” and “fail to stop being on the web” are the key symptoms that may activate other IA symptoms [39]. However, considering the bidirectional relationship between IA and depression, it remains to be examined whether the resulting treatment suits adolescents remains to be examined. Moreover, several cross-sectional studies have been conducted to locate the central symptoms and analyze the comorbidity between IA and depression. For example, a cross-sectional study conducted by Cai et al. found that “anticipation for future online activities” was the most common bridge symptom that linked depression and IA among Chinese adolescents in Macau [40]. In another longitudinal study, “guilty” (i.e., a symptom of depression) and “escape” (i.e., a symptom of IA) were identified as the bridge symptoms in the comorbidity networks of depression and IA over time [41]. Although the aforementioned studies have attempted to uncover the relationship between depression and IA symptoms from both cross-sectional and longitudinal perspectives, none of them have specifically targeted and examined depressed adolescents to explore the symptoms of their IA and the trajectory of these symptoms over time.

Drawing inspiration from network analysis methodology, the present study aimed to explore the effects of online learning on secondary school students’ depression and identify changes in IA symptoms caused by online learning among students who were depressed at the beginning of online learning. This endeavor aimed to offer clinical counselors a more accurate understanding when intervening with this specific population. To achieve this, we compared the prevalence rate of depression at the start and after the end of online learning. Moreover, we utilized a cross-lagged panel network (CLPN) framework to investigate the autoregressive and cross-lagged trajectories of IA symptoms over time among depressed students. Our study focused on the dynamic fluctuations in IA symptoms experienced by Chinese secondary school students during the extended period of online learning amidst the COVID-19 pandemic. Through our research, we strive to provide practical recommendations for appropriate treatments and policies.

## 2. Methods

### 2.1. Participants

The study involved two waves of data collection from middle and high school students in Harbin. The first wave was obtained in February 2022 from 10,104 students using an online questionnaire program on their smartphones, and the second wave was collected in June 2022 using the same method from 8390 students who had been taking online courses for four months. Specifically, due to the spread of COVID-19, students were only able to study online from home, rendering traditional in-person classroom instruction unavailable. Therefore, the entire period from February 2022 (i.e., the beginning of online learning) to June 2022 (i.e., the end of online learning) was dedicated to online learning. By matching the datasets from both waves based on students’ phone numbers, a total of 2415 students (47.9% female, *Mean*_age_ = 14.27, *SD*_age_ = 1.53, range from 10 to 18) were ultimately matched. For the purpose of our study, we used the Patient Health Questionnaire-9 [42] to screen for students with potential depressive tendencies at the baseline, using a cutoff score of 8 [43]. At the baseline, 14.16% (N = 342) of participants had a depressive tendency, and 17.64% (N = 426) participants had a depressive tendency at the follow-up. Finally, data from only 342 students were retained for the final data analysis based on the present study’s purpose.

All students, along with their parents, were provided with signed informed consent before participating. The ethical committee of *** University reviewed and approved the research protocol, with reference number 202112220085.

### 2.2. Measures

#### 2.2.1. The Internet Addiction Test (IAT-20)

The Internet Addiction Test (IAT) was developed by [44] as a tool to assess Internet addiction behaviors. It consists of 20 Likert-style questions that measure the severity of adverse consequences resulting from excessive Internet use. Participants rate each item on a scale of 1 (“not at all”) to 5 (“always”), with higher scores indicating more severe addiction symptoms. The Chinese version of the IAT was validated by Chang and Law in 2008 [45], and previous studies have demonstrated its good reliability and validity [46]. In the current study, the IAT-20 exhibited high internal consistency, with Cronbach’s alpha values of 0.94 in the first wave and 0.96 in the second wave.

#### 2.2.2. Patient Health Questionnaire (PHQ-9)

The PHQ-9 is a nine-item questionnaire that assesses depression symptoms [42]. Each item is rated on a scale from 0 (not at all) to 3 (nearly every day), resulting in a total score ranging from 0 to 27. The Chinese version of the PHQ-9 has been validated as an effective screening tool for depression in the general Chinese population [47]. In the present study, the PHQ-9 demonstrated a high internal consistency (*α* = 0.88).

### 2.3. Statistical Analysis

We used R version 4.2.1 [48] to perform all analyses. *Means*, *standard deviations* (*SD*s), *skewness*, and *kurtosis* of all item scores were inspected.

#### 2.3.1. Cross-Lagged Panel Network Estimation

To investigate the connections between the first and second assessments over time, a cross-lagged panel network (CLPN) analysis was performed using the *glmnet* package [49]. The CLPN method allows visualizing how a single node (i.e., symptom) at the first assessment predicts other nodes at the second assessment while controlling for all other variables at the first assessment. The network consists of directed edges that represent autoregressive coefficients, with arrows pointing to the same node, and cross-lagged coefficients, with arrows pointing to other nodes. The color of the arrows indicates the directionality of the effect, with green indicating positive effects and red indicating negative effects, while the thickness of the lines indicates the strength of the association. We utilized the least absolute shrinkage and selection operator (LASSO) approach to simplify the network by eliminating small regression coefficients. The *qgraph* package [50] was used to visualize all network structures.

In the directed CLPNs, we calculated two centrality indices: “in expected influence” (IEI) and “out expected influence” (OEI). IEI represents the extent to which other symptoms predict one symptom and is calculated as the sum of the values of incoming edges associated with one symptom. On the other hand, OEI represents the degree to which one symptom can predict other symptoms and is calculated as the sum of the values of outgoing edges associated with one symptom.

Since Internet addiction behaviors vary significantly among adolescents [51], we included age and gender as covariates in the cross-lagged panel network.

#### 2.3.2. Network Comparison

To evaluate the edge invariance (i.e., the distributions of edge weights) and global invariance (i.e., the sum of all edge weights) between two networks, we utilized the network comparison test (NCT) with the R package *NetworkComparisonTest* 2.2.1 [52].

#### 2.3.3. Network Stability and Accuracy

To evaluate the stability of the centrality indices, we employed the case-dropping bootstrap approach described by Epskamp et al. [53]. This method provides a correlation stability coefficient (*CS-C*), which represents the maximum proportion of the sample size that can be removed while maintaining a correlation coefficient of 0.7 or higher with a 95% probability using the original sample’s centrality indices. Generally, a *CS-C* of ≥0.25 is considered acceptable. We conducted 1000 iterations of bootstrapped confidence intervals (95% *CIs*) to assess the accuracy of edges, where narrower *CIs* indicate a more precise network. Additionally, we performed bootstrap tests (1000 iterations) based on 0.95 *CIs* to compare differences between edge weights and centrality indices. If the *CIs* did not include zero, it suggested a statistical difference between two edges or nodes. All analyses were carried out using the R package *bootnet* 1.4.3 [53].

### 2.4. Sensitivity Analysis

In our study, we aimed to investigate the effect of random data loss on our main findings. To achieve this goal, we conducted a symptom network analysis to construct an Internet addiction symptom network using two distinct data sets. These data sets, consisting of 7748 and 6034 data points, were not matched in the first and second waves, respectively. Additionally, we then compared these results with the analysis of a subset of matched data points, which included 2356 observations. To do so, we generated weighted connected edge coefficient matrices for each network and analyzed their correlations. Building upon prior research [54], we concluded that if the symptom network structure of the matched data exhibited a high correlation with the symptom network structure of the respective wave of unused data, we could assert the robustness of our main findings.

## 3. Result

### 3.1. Descriptive Analysis

The mean and standard deviation of the IAT-20 items are reported in Table 1. All scores in wave 1 were higher than in the second wave.

### 3.2. Cross-Lagged Panel Network

**Figure 1:**
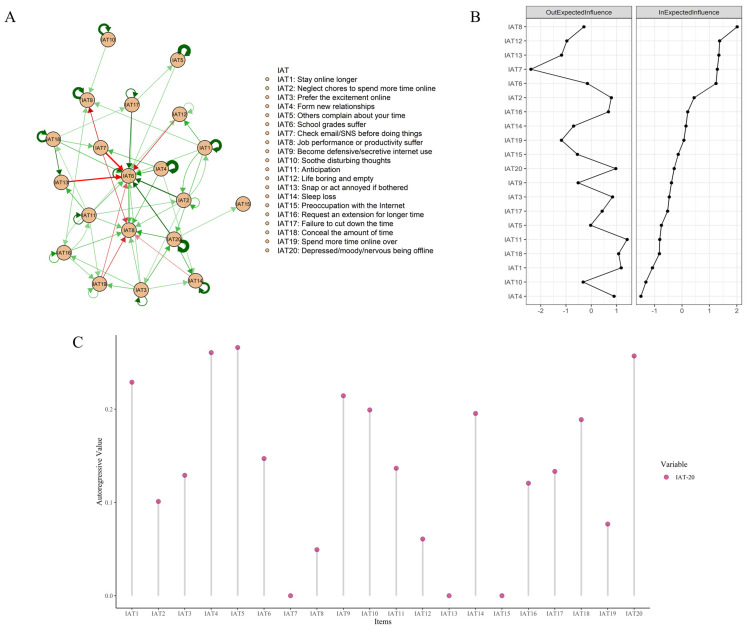
The cross-lagged panel network structure and centrality indexes. (**A**) The network structure. (**B**) Centrality indices (OEI and IEI) of all items. (**C**) Autoregressive coefficients for each item.

In total, 140 edges were not zero (35%) among 400 possible edges, and the edge weight is shown in LASSO cross-lagged regression matrix in Table S1. The edge of “Check email/SNS before doing things”—“School grades suffer” (IAT7–IAT6) showed the strongest cross-lagged association, followed by the edge of “Snap or act annoyed if bothered”—“School grades suffer” (IAT13–IAT6), the edge of “Neglect chores to spend more time online”—“School grades suffer” (IAT2–IAT6) and the edge of “Depressed/moody/nervous being offline”—“School grades suffer” (IAT20–IAT6). See part A of Figure 1.

Part B in Figure 1 showed the CLPN for symptoms for the first time, predicting follow-up symptoms with the centrality plots for OEI and IEI. “Anticipation” (IAT11) had the highest node OEI, followed by “Stay online longer” (IAT1) and “Conceal the amount of time” (IAT18). “Job performance or productivity suffer” (IAT8) had the highest node IEI, followed by “Life boring and empty” (IAT12), “Snap or act annoyed if bothered” (IAT13), “Check email/SNS before doing things” (IAT7), and “School grades suffer” (IAT6). Part C of Figure 1 shows the autoregressive coefficients for each node.

### 3.3. Network Comparison Test

**Figure 2:**
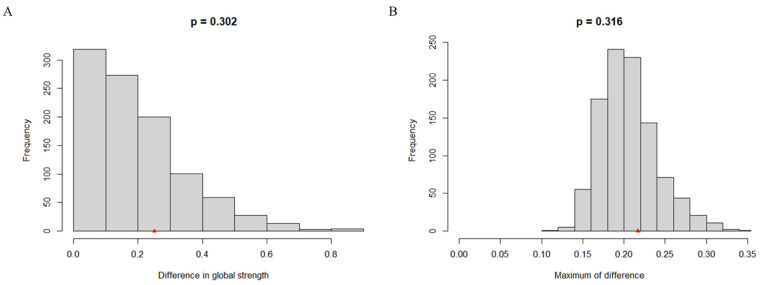
Longitudinal NCT results. (**A**) Network global invariance test. (**B**) Network edge invariance test.

NCT revealed the network structure invariance and global strength invariance, shown in Figure 2. Specifically, there was no significant difference in global network strength (9.11 vs. 9.36, *S*_diff_ = 0.25, *p* = 0.30) and network structure (*M* = 0.22, *p* = 0.32) between wave 1 and wave 2.

### 3.4. Network Accuracy and Stability

**Figure 3:**
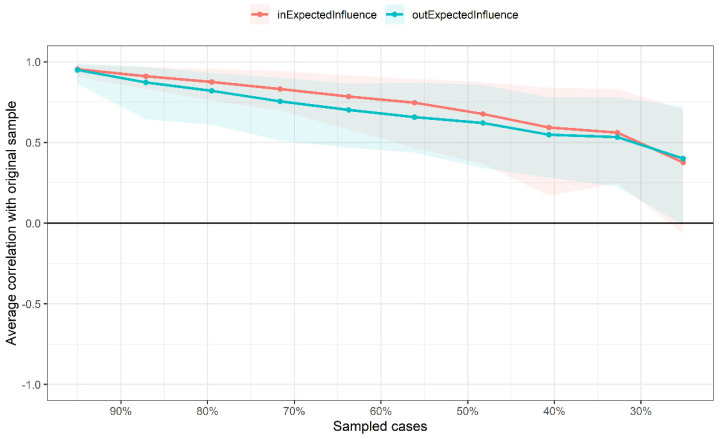
Case-dropping bootstrap test of centrality indices. The x-axis indicates the percentage of cases of the original sample included at each step. The y-axis indicates the correlations between the OEI/IEI from the original network and the OEI/IEI from the networks re-estimated after excluding increasing percentages of cases.

In Figure 3, the cross-lagged network’s case-dropping results indicate that the *CS-C* values of OEI and IEI were 0.13 and 0.28. Furthermore, 95% of bootstrapped *CIs* of edges were narrow (Figure S1), suggesting that edges were trustworthy. Additionally, results of the nonparametric bootstrap procedure revealed that most comparisons among edge weights and centrality indicators were statistically significant (Figures S2–S4).

### 3.5. Sensitivity Analysis

Upon examining the random data loss in the first and second waves, this study discovered that the network structure matrices obtained from the lost 7689 data in the first wave and 5975 data in the second wave were highly similar to the network structure matrix of the matched data. The correlation coefficients between the lost data and matched data were *r* = 0.80 (*p* < 0.001) and *r* = 0.67 (*p* < 0.001) for the first and second waves, respectively.

## 4. Discussion

To begin with, our study found that the prevalence rate of depression increased from 14.16% to 17.64% after four months of online learning. This finding aligns with a previous study that also confirmed the potential for online learning to contribute to a higher incidence rate of depression and anxiety [29]. However, we also noticed that 51.46% of students with depressive tendencies at the beginning of online learning no longer exhibited depressive tendencies after four weeks following the end of online learning. This finding is interesting and consistent with the theory of compensatory Internet use [28]. During the online learning period, students have increased access to the Internet, which can fulfill some of their mental needs and alleviate negative emotions [28].

Meanwhile, our study employed cross-lagged panel analysis to examine the IA network among adolescents with depressive tendencies. In this section, firstly, we identified symptoms with high OEI and IEI. Secondly, we also identified several key directed relationships between different symptoms of IAT before and after long-term online teaching. Thirdly, we compared the differences between the networks of the two waves, aiming to examine the invariance of the network structure and the global strength between the two networks.

Regarding the results of OEI, our research reveals that “Anticipation”, which refers to the expectation of going online again [55], exhibits the highest OEI. This finding suggests that it is the primary symptom in the network that can predict other symptoms, which aligns with previous research. A study categorized adolescents into divorced and non-divorced group based on their parents’ marital status and found that “Anticipation” had the highest OEI in the non-divorced group [46]. Additionally, Cai et al. conducted a study involving 1009 adolescents and constructed a symptom network of depression and IA, where they identified “Anticipation” as a key bridge symptom in the comorbidity network of depression and IA [40]. Future studies can further explore the role of “Anticipation” and the mechanisms by which different symptoms interact with each other in the network.

The most obvious finding to emerge from this study is the prominent role of “School grades suffer”, which indicates a decline in academic performance due to Internet use [56]. We observed that “School grades suffer” is one of the symptoms with high IEI, suggesting that it is frequently influenced by other symptoms. Specifically, several symptoms are directly linked to “School grades suffer”. In wave 1, “Check email/SNS before doing things” and “Snap or act annoyed if bothered” in wave 1 negatively predicted the “School grades suffer” in wave 2. One possible explanation for these results is that the data in wave 1 were collected at the beginning of the holiday, when adolescents had more opportunities to use smartphones, which may contribute to a high level of IA [57]. Consistently, previous research also showed that using the Internet during the holiday would cause a higher level of IA than using the Internet on weekdays [58]. Thus, we can infer that adolescents develop a higher level of IA during holidays due to increased Internet usage frequency. However, online classes, which occupy adolescents’ daily time, may decrease their frequency of playing online games and watching short videos. Consequently, this reduction may contribute to an improvement in students’ academic performance [59,60].

Additionally, we have found that “Neglect chores to spend more time online” and “Depressed/moody/nervous being offline” in wave 1 positively predict “School grades suffer”. “Neglect chores to spend more time online” refers to individuals spending excessive time online instead of engaging in real-life social interactions, while the scores of “Depressed/moody/nervous being offline” reflect negative emotions experienced when offline [61]. We can interpret these results from the perspective of social interaction. During adolescence, peer relationships hold great significance, and numerous studies have demonstrated the association between depression and difficulties in forming close friendships [62,63,64]. According to the compensatory Internet use theory, individuals turn to the Internet as a means to escape difficult situations or negative emotions in real life [65]. Consequently, adolescents with depressive tendencies may face challenges in social interactions and resort to using the Internet as a coping strategy to avoid these difficulties and negative emotions. However, relying on the Internet as an escape mechanism is maladaptive, as it not only intensifies depressive feelings [66] but also hinders their ability to effectively interact with people in real life [67,68], thus perpetuating a negative cycle of using the Internet to escape reality. This significant finding offers valuable insights, suggesting that interventions targeting IA among students with depressive tendencies should focus on helping them develop the skills to build and maintain close relationships in real life. It is important to note that variables such as peer relationships and Internet usage motivation were not measured in our study. Future research should include these variables to further examine the proposed inferences.

As for the network comparison, surprisingly, no differences were found in the network structure or global strength between the two-time points networks. This finding may be attributed to the stability of depression among adolescents. However, based on the t-test results for IAT symptoms scores, all symptoms showed lower scores after four months of online learning. The decrease in scores can be attributed to the end of online learning, as students no longer have unrestricted access to the Internet once they return to school. Therefore, it is important to interpret the null results of the network comparison with caution. Evidence suggests a bidirectional association between Internet use and depression, indicating that depression may contribute to the maintenance or even increase in IA [69,70]. Additionally, a 6-year longitudinal study suggested that adolescents may retain their relative levels of depressive emotions [71], further supporting the notion of relative stability in adolescent depression. Thus, despite finding several significant cross-lagged edges, the stability of depression leads to a stable network structure and global strength over time. This result underscores the need for additional attention to adolescents with depressive tendencies when intervening in IA [72,73,74].

## 5. Limitations and Conclusions

Several limitations in this study need to be noticed. First, network analysis can only provide us with information about the relationship between each symptom but does not reveal the detailed mechanism behind them. Therefore, future studies can design experiments to explore this aspect further. Second, in this study, we used the Internet Addiction Test (IAT-20) to measure the level of IA in adolescents. However, we did not record the time and frequency of Internet use, which may result in a mismatch between the results of the subjective questionnaire and the objective indicators. Additionally, the lack of measurements on specific modalities, such as social networking and video games, further limits our ability to provide detailed explanations. Third, although we screened students with depressive tendencies in this study, they do not represent students with clinical depression, which restricts the generalizability of our findings. Fourth, considering the bidirectional relationship between IA and depression, IA may vary during the onset and remission of depression, which requires further exploration. Finally, as an instrument that measures IA symptoms, IAT is now over 20 years old, and some statements in the questionnaire are simply not applicable today and may yield “false” positive or negative results (e.g., the statement about checking e-mail rather than, for example, WeChat or Facebook and similar platforms). Hence, it is crucial that measurement tools reflecting the current era emerge to assess the symptoms of IA.

Notwithstanding these limitations, to best of our knowledge, this is the first study that utilizes cross-lagged panel network analysis to examine the directed symptom network of IA among adolescents with depressive tendencies. Our research demonstrates that among adolescents with depressive tendencies, “Anticipation” exhibits the highest OEI, while “School grades suffer” shows a high IEI. Furthermore, regarding the cross-lagged associations between different symptoms, our study reveals that “Check email/SNS before doing things” and “Snap or act annoyed if bothered” can negatively predict the “School grades suffer”. In contrast, “Neglect chores to spend more time online” and “Depressed/moody/nervous being offline” positively predict that “School grades suffer”. The current research provides us with information about symptoms of the IAT, highlighting several important symptoms and enhancing our understanding of IA among adolescents with depressive tendencies.

## Figures and Tables

**Table 1:** Descriptive analysis (N = 342).

Variables	*Mean* (*SD*)	*t*	*df*	*p*	Difference [95% *CI*]	Cohen’s *d* [95% *CI*]
IAT1_F	3.15 (1.15)	−3.88	341	<0.001	−0.31 [−0.46, −0.15]	−0.21 [−0.32, −0.10]
IAT1_S	2.84 (1.36)					
IAT2_F	2.80 (1.20)	−3.54	341	<0.001	−0.28 [−0.44, −0.13]	−0.19 [−0.30, −0.09]
IAT2_S	2.51 (1.24)					
IAT3_F	2.60 (1.26)	−2.92	341	0.004	−0.25 [−0.41, −0.08]	−0.16 [−0.26, −0.05]
IAT3_S	2.36 (1.28)					
IAT4_F	2.10 (1.15)	−2.56	341	0.011	−0.18 [−0.32, −0.04]	−0.14 [−0.24, −0.03]
IAT4_S	1.92 (1.14)					
IAT5_F	3.31 (1.28)	−4.92	341	<0.001	−0.40 [−0.56, −0.24]	−0.27 [−0.37, −0.16]
IAT5_S	2.91 (1.39)					
IAT6_F	2.85 (1.24)	−3.24	341	0.001	−0.26 [−0.42, −0.10]	−0.18 [−0.28, −0.07]
IAT6_S	2.58 (1.29)					
IAT7_F	2.28 (1.25)	−2.72	341	0.007	−0.23 [−0.40, −0.06]	−0.15 [−0.25, −0.04]
IAT7_S	2.04 (1.19)					
IAT8_F	2.36 (1.22)	−3.29	341	0.001	−0.26 [−0.41, −0.10]	−0.18 [−0.28, −0.07]
IAT8_S	2.10 (1.21)					
IAT9_F	2.84 (1.40)	−3.44	341	<0.001	−0.31 [−0.49, −0.13]	−0.19 [−0.29, −0.08]
IAT9_S	2.52 (1.43)					
IAT10_F	2.89 (1.38)	−3.90	341	<0.001	−0.34 [−0.51, −0.17]	−0.21 [−0.32, −0.10]
IAT10_S	2.55 (1.37)					
IAT11_F	2.87 (1.24)	−4.89	341	<0.001	−0.40 [−0.56, −0.24]	−0.26 [−0.37, −0.16]
IAT11_ S	2.47 (1.29)					
IAT12_F	2.81 (1.26)	−4.43	341	<0.001	−0.37 [−0.54, −0.21]	−0.24 [−0.35, −0.13]
IAT12_S	2.44 (1.31)					
IAT13_F	2.55 (1.26)	−3.76	341	<0.001	−0.30 [−0.46, −0.14]	−0.20 [−0.31, −0.10]
IAT13_S	2.25 (1.24)					
IAT14_F	2.56 (1.29)	−4.81	341	<0.001	−0.38 [−0.53, −0.22]	−0.26 [−0.37, −0.15]
IAT14_S	2.19 (1.23)					
IAT15_F	2.72 (1.17)	−4.45	341	<0.001	−0.36 [−0.52, −0.20]	−0.24 [−0.35, −0.13]
IAT15_S	2.36 (1.19)					
IAT16_F	2.82 (1.22)	−3.72	341	<0.001	−0.30 [−0.46, −0.14]	−0.20 [−0.31, −0.09]
IAT16_S	2.53 (1.26)					
IAT17_F	2.67 (1.26)	−4.18	341	<0.001	−0.34 [−0.50, −0.18]	−0.23 [−0.33, −0.12]
IAT17_S	2.33 (1.28)					
IAT18_F	2.48 (1.29)	−3.34	341	<0.001	−0.27 [−0.43, −0.11]	−0.18 [−0.29, −0.07]
IAT18_S	2.21 (1.29)					
IAT19_F	2.39 (1.31)	−3.47	341	<0.001	−0.29 [−0.45, −0.12]	−0.19 [−0.29, −0.08]
IAT19_S	2.11 (1.22)					
IAT20_F	2.43 (1.30)	−4.61	341	<0.001	−0.36 [−0.51, −0.20]	−0.25 [−0.36, −0.14]
IAT20_S	2.08 (1.26)					

Note. _F means wave 1. _S means wave 2.

## Data Availability

Analytic code and data for this work are available upon request.

## Supplementary Materials

The following supporting information can be downloaded at: https://www.mdpi.com/article/10.3390/bs13070520/s1, Figure S1: Nonparametric bootstrapped confidence intervals of estimated edges for cross-sectional networks. The red line represents the estimated edge, while the dark area indicates the 95% bootstrap confidence interval; Figure S2: Bootstrapped stability test for cross-lagged edge weights. Figure S3: Nonparametric bootstrapped difference test for each node’s centrality index (inExpectedInfluence). Grey boxes indicate no significant difference, whereas black boxes indicate a statistically significant difference (*p* < 0.05). Figure S4: Nonparametric bootstrapped difference test for each node’s centrality index (outExpectedInfluence). Grey boxes indicate no significant difference, whereas black boxes indicate a statistically significant difference (*p* < 0.05). Table S1: LASSO cross-lagged regression matrix of all IAT items. Each number in the matrix represents the regression coefficient of the item in the same row on its left side (wave 1) predicting the item in the same column on its upper side (wave 2).

## References

[1] Kandell J.J. (1998). Internet addiction on campus: The vulnerability of college students. Cyberpsychol. Behav..

[2] Young K.S. (1999). The research and controversy surrounding internet addiction. Cyberpsychol. Behav..

[3] Kuss D.J., Griffiths M.D. (2011). Online social networking and addiction—A review of the psychological literature. Int. J. Environ. Res. Public Health.

[4] Triberti S., Milani L., Villani D., Grumi S., Peracchia S., Curcio G., Riva G. (2018). What matters is when you play: Investigating the relationship between online video games addiction and time spent playing over specific day phases. Addict. Behav. Rep..

[5] Young K.S. (1998). Internet Addiction: The Emergence of a New Clinical Disorder. Cyberpsychol. Behav..

[6] Ha J.H., Kim S.Y., Bae S.C., Bae S., Kim H., Sim M., Lyoo I.K., Cho S.C. (2007). Depression and Internet Addiction in Adolescents. Psychopathology.

[7] Tereshchenko S., Kasparov E. (2019). Neurobiological Risk Factors for the Development of Internet Addiction in Adolescents. Behav. Sci..

[8] Ozturk E., Ozmen S.K. (2016). The relationship of self-perception, personality and high school type with the level of problematic internet use in adolescents. Comput. Hum. Behav..

[9] Cheng C., Li A.Y.-L. (2014). Internet Addiction Prevalence and Quality of (Real) Life: A Meta-Analysis of 31 Nations Across Seven World Regions. Cyberpsychol. Behav. Soc. Netw..

[10] Griffiths M.D., Kuss D.J., Demetrovics Z. (2014). Social Networking Addiction: An Overview of Preliminary Findings.

[11] Whang L.S.M., Lee S., Chang G. (2003). Internet over-users’ psychological profiles: A behavior sampling analysis on Internet addiction. Cyberpsychol. Behav..

[12] Lozano-Blasco R., Robres A.Q., Sánchez A.S. (2022). Internet addiction in young adults: A meta-analysis and systematic review. Comput. Hum. Behav..

[13] Meng S.-Q., Cheng J.-L., Li Y.-Y., Yang X.-Q., Zheng J.-W., Chang X.-W., Shi Y., Chen Y., Lu L., Sun Y. (2022). Global prevalence of digital addiction in general population: A systematic review and meta-analysis. Clin. Psychol. Rev..

[14] Gao M., Teng Z., Wei Z., Jin K., Xiao J., Tang H., Wu H., Yang Y., Yan H., Chen J. (2022). Internet addiction among teenagers in a Chinese population: Prevalence, risk factors, and its relationship with obsessive-compulsive symptoms. J. Psychiatr. Res..

[15] Wu Q., Ren Q., Zhong N., Bao J., Zhao Y., Du J., Chen T., Zhao M. (2022). Internet behavior patterns of adolescents before, during, and after COVID-19 pandemic. Front. Psychiatry.

[16] Canan F., Yildirim O., Sinani G., Ozturk O., Ustunel T.Y., Ataoglu A. (2013). Internet addiction and sleep disturbance symptoms among Turkish high school students. Sleep Biol. Rhythm..

[17] Yayan E.H., Arikan D., Saban F., Gürarslan Baş N., Özel Özcan Ö. (2017). Examination of the correlation between Internet addiction and social phobia in adolescents. West. J. Nurs. Res..

[18] Lee Y.S., Han D.H., Kim S.M., Renshaw P.F. (2013). Substance abuse precedes internet addiction. Addict. Behav..

[19] Yang L., Sun L., Zhang Z., Sun Y., Wu H., Ye D. (2014). Internet addiction, adolescent depression, and the mediating role of life events: Finding from a sample of Chinese adolescents. Int. J. Psychol..

[20] Akin A., Iskender M. (2011). Internet addiction and depression, anxiety and stress. Int. Online J. Educ. Sci..

[21] Khatcherian E., Zullino D., De Leo D., Achab S. (2022). Feelings of Loneliness: Understanding the Risk of Suicidal Ideation in Adolescents with Internet Addiction. A Theoretical Model to Answer to a Systematic Literature Review, without Results. Int. J. Environ. Res. Public Health.

[22] Lau J.T.F., Walden D.L., Wu A.M.S., Cheng K.-M., Lau M.C.M., Mo P.K.H. (2018). Bidirectional predictions between Internet addiction and probable depression among Chinese adolescents. J. Behav. Addict..

[23] McRae K. (2016). Cognitive emotion regulation: A review of theory and scientific findings. Curr. Opin. Behav. Sci..

[24] Joormann J., Quinn M.E. (2014). Cognitive processes and emotion regulation in depression. Depress. Anxiety.

[25] Yildiz M.A. (2017). Emotion regulation strategies as predictors of internet addiction and smartphone addiction in adolescents. J. Educ. Sci. Psychol..

[26] Luszczynska A., Schwarzer R. (2015). Social cognitive theory. Fac. Health Sci. Publ..

[27] Saritepeci M., Yildiz Durak H., Atman Uslu N. (2022). A latent profile analysis for the study of multiple screen addiction, mobile social gaming addiction, general mattering, and family sense of belonging in university students. Int. J. Ment. Health Addict..

[28] Gao T., Li J., Zhang H., Gao J., Kong Y., Hu Y., Mei S. (2018). The influence of alexithymia on mobile phone addiction: The role of depression, anxiety and stress. J. Affect. Disord..

[29] AlAzzam M., Abuhammad S., Abdalrahim A., Hamdan-Mansour A.M. (2021). Predictors of depression and anxiety among senior high school students during COVID-19 pandemic: The context of home quarantine and online education. J. Sch. Nurs..

[30] Arpaci I., Kesici Ş., Baloğlu M. (2018). Individualism and internet addiction: The mediating role of psychological needs. Internet Res..

[31] Deng L.Y., Fang X.Y., Wan J.J., Zhang J.T., Xia C.C. (2012). Psychological Needs, Need Gratification and Internet Addiction among College Students. J. Psychol. Sci..

[32] Li D., Zhou Y., Zhao L., Wang Y., Sun W. (2016). Cumulative ecological risk and adolescent internet addiction: The mediating role of basic psychological need satisfaction and positive outcome expectancy. Acta Psychol. Sin..

[33] Tonioni F., D’Alessandris L., Lai C., Martinelli D., Corvino S., Vasale M., Fanella F., Aceto P., Bria P. (2012). Internet addiction: Hours spent online, behaviors and psychological symptoms. Gen. Hosp. Psychiatry.

[34] Zhang H.-X., Jiang W.-Q., Lin Z.-G., Du Y.-S., Vance A. (2013). Comparison of psychological symptoms and serum levels of neurotransmitters in Shanghai adolescents with and without internet addiction disorder: A case-control study. PLoS ONE.

[35] Borsboom D., Cramer A.O. (2013). Network analysis: An integrative approach to the structure of psychopathology. Annu. Rev. Clin. Psychol..

[36] Borsboom D., Deserno M.K., Rhemtulla M., Epskamp S., Fried E.I., McNally R.J., Robinaugh D.J., Perugini M., Dalege J., Costantini G. (2021). Network analysis of multivariate data in psychological science. Nat. Rev. Methods Prim..

[37] Hirota T., McElroy E., So R. (2021). Network Analysis of Internet Addiction Symptoms Among a Clinical Sample of Japanese Adolescents with Autism Spectrum Disorder. J. Autism Dev. Disord..

[38] Barlow D.H., Durand V.M., Hofmann S.G. (2016). Abnormal Psychology: An Integrative Approach.

[39] Lu J., Zhang Q., Zhong N., Chen J., Zhai Y., Guo L., Lu C., Chen T., Jiang Z., Zheng H. (2022). Addiction Symptom Network of Young Internet Users: Network Analysis. J. Med. Internet Res..

[40] Cai H., Bai W., Sha S., Zhang L., Chow I.H., Lei S.-M., Lok G.K., Cheung T., Su Z., Hall B.J. (2022). Identification of central symptoms in Internet addictions and depression among adolescents in Macau: A network analysis. J. Affect. Disord..

[41] Zhao Y., Qu D., Chen S., Chi X. (2023). Network analysis of internet addiction and depression among Chinese college students during the COVID-19 pandemic: A longitudinal study. Comput. Hum. Behav..

[42] Kroenke K., Spitzer R.L., Williams J.B. (2001). The PHQ-9: Validity of a brief depression severity measure. J. Gen. Intern. Med..

[43] Manea L., Gilbody S., McMillan D. (2012). Optimal cut-off score for diagnosing depression with the Patient Health Questionnaire (PHQ-9): A meta-analysis. CMAJ.

[44] Young K.S. (1998). Caught in the Net: How to Recognize the Signs of Internet Addiction—And a Winning Strategy for Recovery.

[45] Chang M.K., Law S.P.M. (2008). Factor structure for Young’s Internet Addiction Test: A confirmatory study. Comput. Hum. Behav..

[46] Niu H., Wang S., Tao Y., Tang Q., Zhang L., Liu X. (2023). The association between online learning, parents’ marital status, and internet addiction among adolescents during the COVID-19 pandemic period: A cross-lagged panel network approach. J. Affect. Disord..

[47] Tao Y., Hou W., Niu H., Ma Z., Zheng Z., Wang S., Liu X., Zhang L. (2023). Comparing the centrality symptoms of major depressive disorder samples across junior high school students, senior high school students, college students and elderly adults during city lockdown of COVID-19 pandemic—A network analysis. J. Affect. Disord..

[48] Team R.C. (2023). R: A Language and Environment for Statistical Computing, 4.3.0. https://www.r-project.org/.

[49] Friedman J., Hastie T., Tibshirani R. (2010). Regularization paths for generalized linear models via coordinate descent. J. Stat. Softw..

[50] Epskamp S., Cramer A.O., Waldorp L.J., Schmittmann V.D., Borsboom D. (2012). qgraph: Network visualizations of relationships in psychometric data. J. Stat. Softw..

[51] Karacic S., Oreskovic S. (2017). Internet addiction through the phase of adolescence: A questionnaire study. JMIR Ment. Health.

[52] Van Borkulo C.D., van Bork R., Boschloo L., Kossakowski J.J., Tio P., Schoevers R.A., Borsboom D., Waldorp L.J. (2022). Comparing network structures on three aspects: A permutation test. Psychol. Methods.

[53] Epskamp S., Borsboom D., Fried E.I. (2018). Estimating psychological networks and their accuracy: A tutorial paper. Behav. Res. Methods.

[54] Tao Y., Niu H., Hou W., Zhang L., Ying R. (2023). Hopelessness during and after the COVID-19 pandemic lockdown among Chinese college students: A longitudinal network analysis. J. Clin. Psychol..

[55] Lu X., Yeo K.J., Guo F., Zhao Z., Wu O. (2022). Psychometric property and measurement invariance of internet addiction test: The effect of socio-demographic and internet use variables. BMC Public Health.

[56] Cai H., Xi H.-T., An F., Wang Z., Han L., Liu S., Zhu Q., Bai W., Zhao Y.-J., Chen L. (2021). The association between Internet addiction and anxiety in nursing students: A network analysis. Front. Psychiatry.

[57] Ben-Yehuda L., Greenberg L., Weinstein A. (2016). Internet addiction by using the smartphone-relationships between internet addiction, frequency of smartphone use and the state of mind of male and female students. J. Reward Defic. Syndr. Addict. Sci..

[58] ElSalhy M., Miyazaki T., Noda Y., Nakajima S., Nakayama H., Mihara S., Kitayuguchi T., Higuchi S., Muramatsu T., Mimura M. (2019). Relationships between Internet addiction and clinicodemographic and behavioral factors. Neuropsychiatr. Dis. Treat..

[59] Baert S., Vujić S., Amez S., Claeskens M., Daman T., Maeckelberghe A., Omey E., De Marez L. (2020). Smartphone use and academic performance: Correlation or causal relationship?. Kyklos.

[60] Amez S., Baert S. (2020). Smartphone use and academic performance: A literature review. Int. J. Educ. Res..

[61] Lu J.-X., Zhai Y.-J., Chen J., Zhang Q.-H., Chen T.-Z., Lu C.-L., Jiang Z.-L., Guo L., Zheng H. (2023). Network analysis of internet addiction and sleep disturbance symptoms. Prog. Neuro-Psychopharmacol. Biol. Psychiatry.

[62] La Greca A.M., Harrison H.M. (2005). Adolescent peer relations, friendships, and romantic relationships: Do they predict social anxiety and depression?. J. Clin. Child Adolesc. Psychol..

[63] Platt B., Kadosh K.C., Lau J.Y. (2013). The role of peer rejection in adolescent depression. Depress. Anxiety.

[64] Schwartz-Mette R.A., Shankman J., Dueweke A.R., Borowski S., Rose A.J. (2020). Relations of friendship experiences with depressive symptoms and loneliness in childhood and adolescence: A meta-analytic review. Psychol. Bull..

[65] Kardefelt-Winther D. (2014). A conceptual and methodological critique of internet addiction research: Towards a model of compensatory internet use. Comput. Hum. Behav..

[66] Elhai J.D., Dvorak R.D., Levine J.C., Hall B.J. (2017). Problematic smartphone use: A conceptual overview and systematic review of relations with anxiety and depression psychopathology. J. Affect. Disord..

[67] Dwyer R.J., Kushlev K., Dunn E.W. (2018). Smartphone use undermines enjoyment of face-to-face social interactions. J. Exp. Soc. Psychol..

[68] Ran G., Li J., Zhang Q., Niu X. (2022). The association between social anxiety and mobile phone addiction: A three-level meta-analysis. Comput. Hum. Behav..

[69] Gámez-Guadix M. (2014). Depressive symptoms and problematic Internet use among adolescents: Analysis of the longitudinal relationships from the cognitive–behavioral model. Cyberpsychol. Behav. Soc. Netw..

[70] Yang X., Guo W.-J., Tao Y.-J., Meng Y.-J., Wang H.-Y., Li X.-J., Zhang Y.-M., Zeng J.-K., Tang W.-J., Wang Q. (2022). A bidirectional association between internet addiction and depression: A large-sample longitudinal study among Chinese university students. J. Affect. Disord..

[71] Holsen I., Kraft P., Vittersø J. (2000). Stability in depressed mood in adolescence: Results from a 6-year longitudinal panel study. J. Youth Adolesc..

[72] Cheung J.C.-S., Chan K.H.-W., Lui Y.-W., Tsui M.-S., Chan C. (2018). Psychological well-being and adolescents’ internet addiction: A school-based cross-sectional study in Hong Kong. Child Adolesc. Soc. Work J..

[73] Lee J.Y., Shin K.M., Cho S.-M., Shin Y.M. (2014). Psychosocial risk factors associated with internet addiction in Korea. Psychiatry Investig..

[74] Yen J.Y., Ko C.H., Yen C.F., Chen S.H., Chung W.L., Chen C.C. (2008). Psychiatric symptoms in adolescents with Internet addiction: Comparison with substance use. Psychiatry Clin. Neurosci..

