# Supplementary material for: The Impact of Long-Term Online Learning on Internet Addiction Symptoms among Depressed Secondary School Students: Insights from a Cross-Panel Network Analysis
Source: Behav Sci (Basel). 2023 Jun 21;13(7):520. doi: 10.3390/bs13070520 (PMC10376411; doi:10.3390/bs13070520)
Supplement: Supplementary file 1 [file behavsci-13-00520-s001.zip › behavsci-2434546-supplementary.pdf]

Table S1. LASSO cross-lagged regression matrix of all IAT items. Each number in the matrix represents the regression coefficient of the item in the same row on its left side (wave 1) predicting the item in the same column on its upper side (wave 2).

	IAT1	IAT2	IAT3	IAT4	IAT5	IAT6	IAT7	IAT8	IAT9	IAT10	IAT11	IAT12	IAT13	IAT14	IAT15	IAT16	IAT17	IAT18	IAT19	IAT20
1	0.23	0.09	0.01	0.00	0.00	0.06	0.01	0.02	0.06	0.04	0.00	0.10	0.03	0.03	0.00	0.00	0.04	0.00	0.00	0.08
2	0.07	0.10	0.05	0.00	0.01	0.14	0.00	0.07	0.00	0.00	0.02	0.00	0.05	0.01	0.00	0.05	0.00	0.02	0.00	0.03
3	0.00	0.00	0.13	0.00	0.00	0.06	0.00	0.07	0.00	0.00	0.00	0.00	0.00	0.06	0.02	0.07	0.00	0.00	0.08	0.10
4	0.00	0.00	0.00	0.26	0.00	0.12	0.09	0.09	0.02	0.00	0.00	0.04	0.00	0.05	0.00	0.00	0.00	0.00	0.00	0.00
5	0.00	0.00	0.04	0.00	0.27	0.06	0.00	0.02	0.00	0.00	0.00	0.00	0.00	0.00	0.01	0.04	0.00	0.03	0.00	0.00
6	0.00	0.00	0.01	0.00	0.00	0.15	0.04	0.09	0.00	0.00	0.00	0.00	0.00	0.00	0.00	0.00	0.00	0.00	0.00	0.00
7	0.00	0.00	0.00	0.00	0.00	(0.16)	0.00	(0.09)	(0.12)	0.00	0.00	0.00	0.00	(0.05)	0.00	0.00	0.00	0.00	0.00	(0.05)
8	0.00	0.00	0.00	0.00	0.01	(0.02)	0.00	0.05	0.04	0.00	0.00	0.00	0.00	0.00	0.00	0.00	0.03	0.00	0.00	0.00
9	0.00	0.00	0.00	0.00	0.00	0.00	0.01	0.00	0.21	0.00	0.00	0.00	0.00	0.00	0.00	0.00	0.00	0.00	0.00	0.00
10	0.00	0.00	0.00	0.00	0.00	0.00	0.00	0.00	0.06	0.20	0.00	0.00	0.00	0.00	0.00	0.00	0.00	0.00	0.00	0.00
11	0.00	0.06	0.00	0.00	0.03	0.08	0.03	0.07	0.00	0.00	0.14	0.05	0.10	0.00	0.04	0.05	0.00	0.05	0.06	0.00
12	0.00	0.00	0.00	0.00	0.00	(0.11)	0.00	0.00	0.00	0.00	0.00	0.06	0.00	0.00	0.00	0.00	0.00	0.00	0.00	(0.01)
13	0.00	0.00	0.00	0.00	0.00	(0.15)	0.00	(0.02)	0.00	0.00	0.00	0.00	0.00	0.00	0.00	0.00	0.00	0.00	0.00	0.00
14	0.00	0.00	0.00	0.00	0.00	(0.02)	0.00	(0.06)	0.00	0.00	0.00	0.01	0.00	0.20	0.02	0.00	0.00	0.00	0.00	0.00
15	0.00	0.00	0.00	0.00	0.00	0.00	0.00	0.00	0.00	0.00	0.00	0.00	0.00	0.00	0.00	0.00	0.00	0.00	0.00	0.00
16	0.00	0.01	0.00	0.00	0.00	0.00	0.04	0.08	0.06	0.00	0.06	0.03	0.04	0.00	0.00	0.12	0.00	0.02	0.07	0.01
17	0.00	0.01	0.00	0.00	0.06	0.13	0.05	0.07	0.00	0.00	0.00	0.00	0.01	0.00	0.00	0.00	0.13	0.00	0.00	0.00
18	0.00	0.04	0.00	0.00	0.00	0.10	0.07	0.04	0.00	0.00	0.01	0.00	0.12	0.00	0.04	0.00	0.05	0.19	0.00	0.02
19	0.00	0.00	0.04	0.00	0.00	(0.08)	0.00	(0.10)	0.00	0.00	0.00	0.00	0.00	0.00	0.00	0.01	0.00	0.00	0.08	0.00
20	0.00	0.00	0.00	0.00	0.00	0.14	0.00	0.11	0.00	0.00	0.03	0.04	0.01	0.11	0.06	0.00	0.00	0.00	0.00	0.26

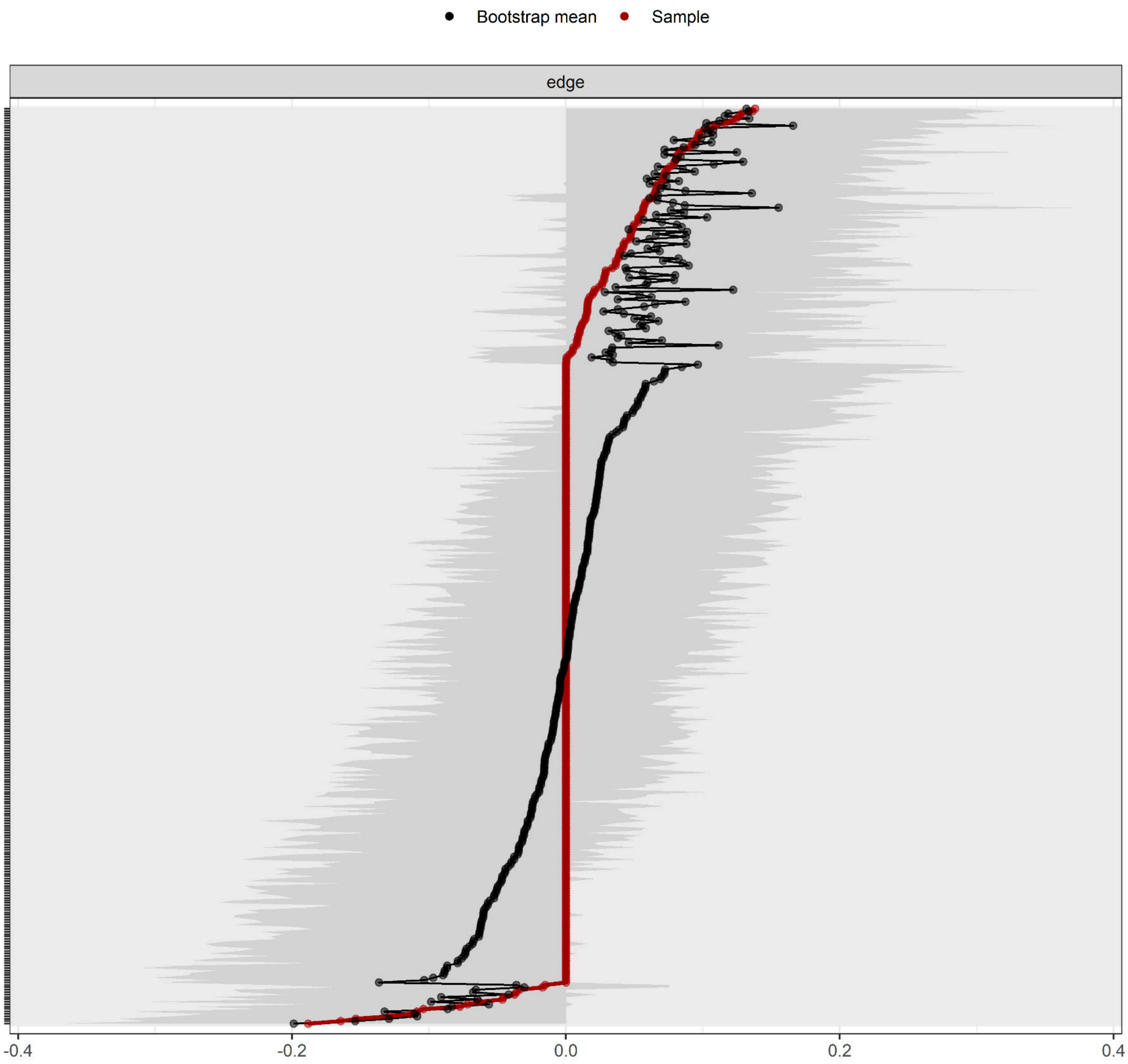

Figure S1. Nonparametric bootstrapped confidence intervals of estimated edges for cross-sectional networks. The red line represents the estimated edge, while the dark area indicates the 95% bootstrap confidence interval.

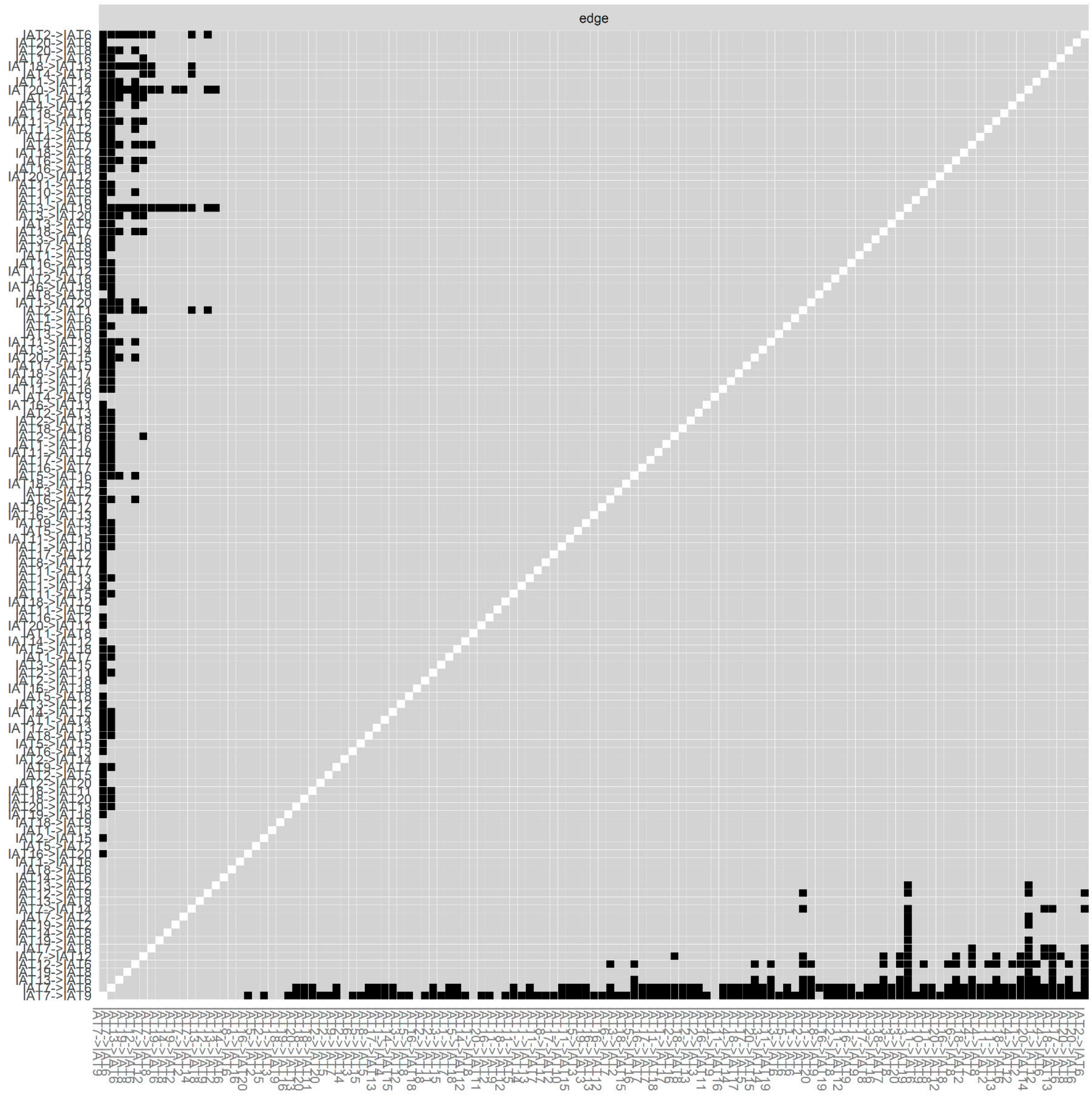

Figure S2. Bootstrapped stability test for cross-lagged edge weights.

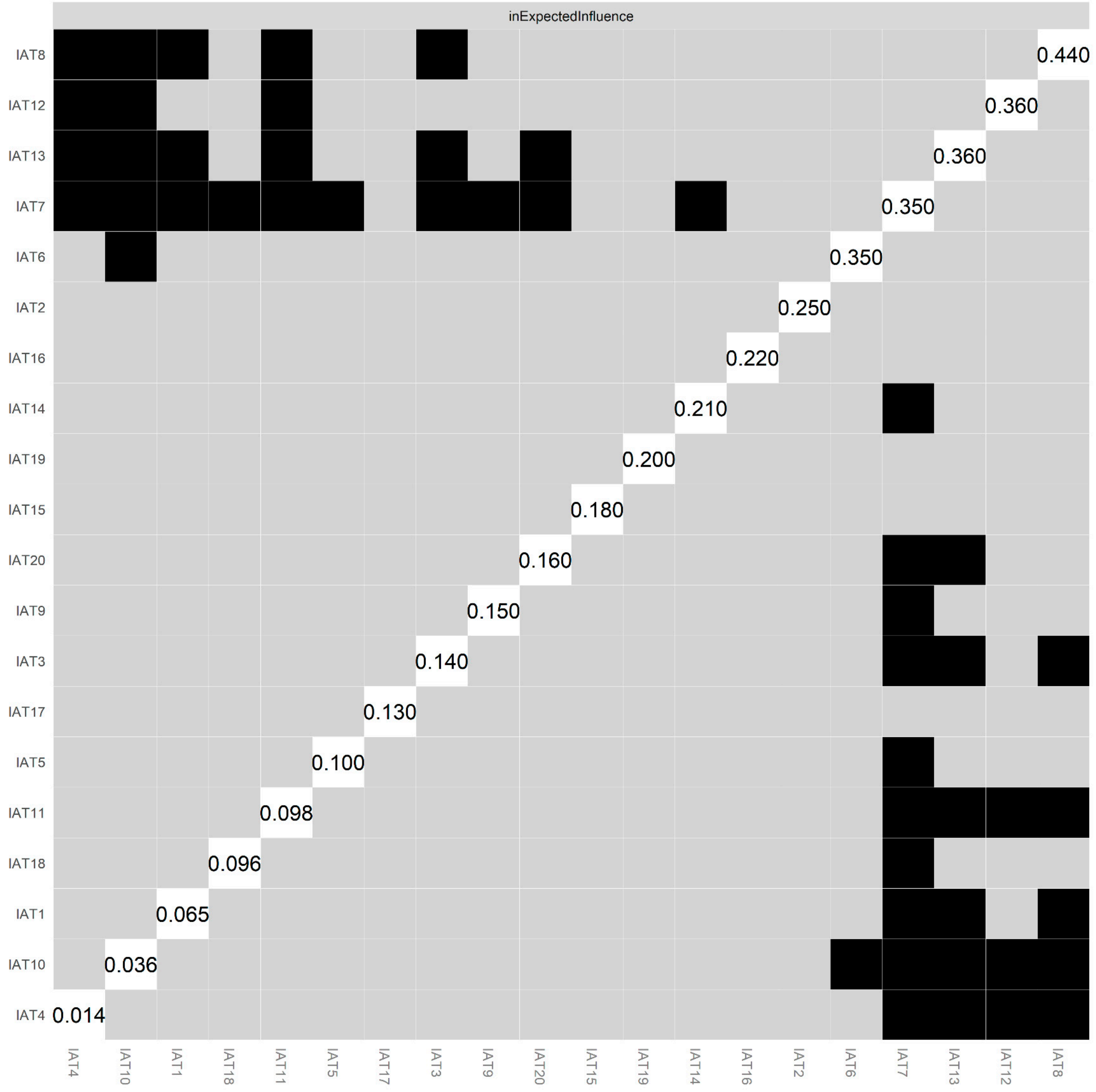

Figure S3. Nonparametric bootstrapped difference test for each node's centrality index (inExpectedInfluence). Grey boxes indicate no significant difference, whereas black boxes indicate a statistically significant difference ($p < 0.05$).

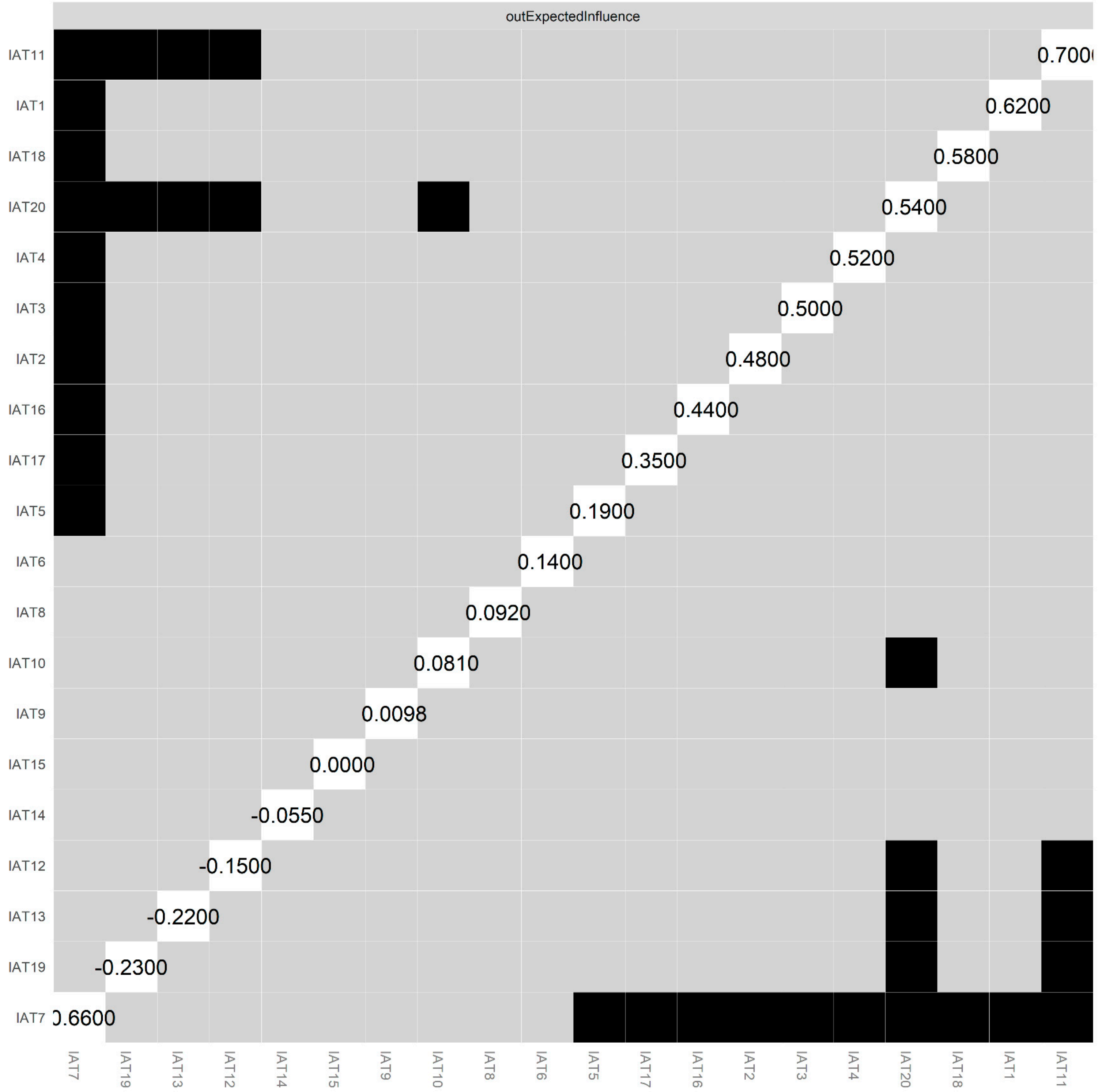

Figure S4. Nonparametric bootstrapped difference test for each node's centrality index (outExpectedInfluence). Grey boxes indicate no significant difference, whereas black boxes indicate a statistically significant difference ($p < 0.05$).
